# Continuous football player tracking from discrete broadcast data

**DOI:** 10.1098/rsos.251175

**Published:** 2025-10-22

**Authors:** Matthew J. Penn, Christl A. Donnelly, Samir Bhatt

**Affiliations:** ^1^ Department of Statistics, University of Oxford, Oxford, UK; ^2^ Pandemic Sciences Institute, Oxford University, Oxford, Oxfordshire, UK; ^3^ Section of Epidemiology, University of Copenhagen, Kobenhavn, Denmark; ^4^ MRC Centre for Global Infectious Disease Analysis, Imperial College London, London, UK

**Keywords:** football, tracking, time series, forecasting

## Abstract

Player tracking data remain out of reach for many professional football teams, as their video feeds are not sufficiently high quality for computer vision technologies to be used. To help bridge this gap, we present a method that can estimate continuous full-pitch tracking data from discrete data made from broadcast footage. Such data could be collected by clubs or players at a similar cost to event data, which are widely available down to the semi-professional level. We test our method using open-source tracking data and include a version that can be applied to a large set of over 200 games with such discrete data.

## 1. Introduction

Data analysis is playing an increasingly important role for a growing number of football clubs at a wide range of levels [1,2]. A key driver underpinning this growth is the availability of match event data—a set of events that describe the on-ball actions in a football match. Because it can be collected from broadcast footage through manual processing, event data are available for an increasingly large number of divisions, allowing a wide range of professional and semi-professional clubs to invest in obtaining and understanding the insights it provides. Crucial to this availability is the fact that the amount of event data per match is sufficiently small for it to be feasibly produced from match video by humans [3]—something that is necessary at lower levels of football, as the quality of the footage is generally too low to enable computer vision algorithms to automate the process.

However, the usefulness of event data is limited by the lack of context they provide for each action. The gold standard of match data is player tracking data [4], where the position of each player (and the ball) is provided at high frame rates, and access to this remains limited. Producing these data by hand would be incredibly time-consuming, and many clubs will not record the whole pitch but will instead capture only the area close to the ball, meaning that a number of players are not visible, so even manual input would lead to partially incomplete datasets. Moreover, open-source player tracking data are extremely sparse, with only around 20 available matches, making it difficult for researchers and smaller football clubs to train and develop models without purchasing expensive datasets.

To seek to remedy this problem, we introduce a novel method for estimating continuous, full-match tracking data from highly discrete, incomplete player positions, where only the team, but not the name of each player, is recorded in each frame. We believe that creating such a dataset would be possible in a similar amount of time to event data, and industry examples already exist, such as Statsbomb’s 360 data [5]. By providing code that can process Statsbomb 360 matches, we also enable researchers to access approximate continuous tracking datasets for around 200 matches [6].

There is a range of previous work that focuses on the creation of sports player tracking data. The focus in these studies is wide, including those which focus on getting player trajectories directly from video footage [7–9], predicting player trajectories using their past positions [10,11] or making predictions using players’ postures [12]. More closely related to our work, [13] examines a similar problem to the one we consider, attempting to predict player trajectories from broadcast data. However, these predictions were only made during short time intervals (9.5 s) and using high-frequency input data (at 6.25 frames per second) and , therefore, would not be applicable to our problem.

The authors of [14] seek to address a similar issue to this paper by building a model that predicts all players’ positions using only event data. This provides only one location per data point (equating to each player’s position being recorded, on average, around once every 30 s), but provides scope for some positional analysis to be carried out on any current set of event data. However, as we will show later, our method provides a middle ground between full tracking data and this event-data-based approximation, with the extra data enabling us to achieve better results than [14], even when we compare our off-camera predictions to their overall error.

The natural approach to this problem (used in, for example, [13] and [14]) would be to make our prediction model using a graph neural network. However, because of the sparsity of open-source training data (we use two matches compared to the 34 in [14] and 105 in [13], which were acquired directly from football data providers), and our aim to make a completely open-source model, we instead use a more parsimonious model, building a comparatively simple model based on autoregressive methods. This still provides a robust and useful prediction model, but we expect that it could be substantially improved upon with access to more data.

## 2. Methods

### 2.1. Summary

The overall method is as follows. Firstly, we will build a predictive model for a player’s position over time based on their past positions and the ball position. Using this model, we will create player trajectories by assigning each of the set of observed player positions in each frame to a trajectory. Once this has been done, we will then build a model to interpolate between the points in each trajectory to create continuous player paths.

Note that the aim of this code is not to track individual player trajectories, but to simply provide the positions of the players on each team. While tracking individual trajectories would be preferable (and is possible on short timescales), events such as corners—where players move, off-camera, from a variety of locations to all be in a very small area—mean that it is impossible to reliably track individual players over the course of the match. This could be remedied by labelling each player whose position was recorded with their name, although this would require substantial additional effort from the creators of the data.

### 2.2. Notation and preliminaries

We suppose that the frames are sampled at times $t_{1},...,t_{n}$ and that the ball position, $b(t_{i})$ *is* known at each frame i.

We assume throughout that the team, and whether or not they are an outfielder or a goalkeeper, of each player in the frame has also been provided, but that the specific identity of a player is not given. Such classification can be done easily using the shirt colour of the players and is provided in datasets such as the Statsbomb 360 data [5]. Thus, we can separately estimate the trajectories of the players on each team (though our final interpolation stage will involve the players from both teams).

Following the convention used in the Statsbomb data, we assume that the pitch measures 120 m by 80 m. This means that when using the Metrica Sports player tracking data [15], where coordinates are given as percentages (i.e. the centre spot is (0.5,0.5) and the goals are (0,0.5) and (1,0.5)), we scale them to ensure they are comparable.

### 2.3. Player position forecasting

Suppose that we know the past trajectory $X=\left\{(x(t_{1}),t_{1}),...,(x(t_{m}),t_{m})\right\}$ up to time $t_{m}$ of a given player, where $x(t_{i})$ *is* the position at time $t_{i}$. Note that we may not necessarily know the position of the player at every sampled frame.

We aim to define functions μ(t,b,X) and σ(t,b,X) such that our predicted trajectory x(t) for $t>t_{m}$ is


(2.1) $$x(t)∼\text{N}(\mu (t,b,X),\sigma (t,b,X{)}^{2}I),$$

where I is the 2 × 2 identity matrix. Note that we will use the position of the ball after time $t_{m}$, as well as the positions before it.

We do this by building a discrete autoregressive moving average with exogenous inputs (ARMAX) model for player positions, using the ball position as an exogenous variable (as players will, in general, move in a similar direction to the ball). In the method presented in this section, we will assume that we use a temporal grid size of 1 s, but other sizes could be used. We use $x^{k}$ to denote the (estimated) position at time k. For $t_{i}<k<t_{i+1}$, we can estimate the value of $x^{k}$ using a simple linear interpolation


(2.2) $$\begin{array}{lcr} & x^{k}=x(t_{i})+\frac{k-t_{i}}{t_{i+1}-t_{i}}(x(t_{i+1})-x(t_{i})). & \end{array}$$

We train our ARMAX model using player trajectories taken from Match 2 of the Metrica Sports open dataset [15] (we reserve Match 1 for testing our model). With more data, it would be possible to train separate models for different positions (as, for example, a left back would show far different movement to a right winger), but given the relatively small size of the data, we simply append all outfield player trajectories together to train a single ARMAX model.

When we predict $x^{k+n}$ from the points $x^{1},...,x^{k}$*,* we use the ARMAX model only when n>1. For n=1, we instead suppose that $x^{k+1}=N(x^{k},s^{2}I)$ for some constant s. For players such as central defenders with smaller responses to the ball movement, this prevents inaccurate estimates, causing incorrect trajectory assignment.

### 2.4. Trajectory assignment

For each of the 10 outfield players on a given team, we aim to build trajectories of known points (i.e. of player positions that are recorded in a frame). We suppose that the trajectory $T_{i}$ of player i *is* given by


(2.3) $$\begin{array}{lcr} & T_{i}=\{(x_{i}(t_{1}^{i}),t_{1}^{i}),...,(x_{i}(t_{m_{i}}^{i}),t_{m_{i}}^{i})\}. & \end{array}$$

At each frame, we append each of the N visible player positions to one of these trajectories (and must ensure that no trajectory has multiple positions from the same frame appended to it, as a player cannot simultaneously be in more than one position). Using our predictive model, the position of player i at the frame time t is given by


(2.4) $$x_{i}(t)∼\text{N}(\mu (t,b,T_{i}),\sigma (t,b,T_{i}{)}^{2}I),$$

and we can therefore define a matrix Q by


(2.5) $$\begin{array}{lcr} & Q_{ij}=\log(\mathbb{P}(\text{player }i\text{ is at visible position }j\text{ at time }t)). & \end{array}$$

The maximum likelihood assignment of players to points can then be found using the Hungarian algorithm on the matrix −Q (as, treating $-Q_{ij}$ as the cost of assigning position j to trajectory i, minimizing the cost is the same as maximizing the sum of the log-likelihoods and therefore the overall log-likelihood).

### 2.5. Player position initialization

Calculating the matrix $Q_{ij}$ requires some previous information on the location of the players. Generally, the majority of outfield players are visible in the initial frame. Those that are not visible are assigned to plausible positions along the defensive line. This may lead to some errors early on in the fitting procedure, but we have found these to be generally insubstantial.

### 2.6. Continuous path interpolation

Given the final processed trajectories $T_{i}$, we then aim to create a continuous path from the points it contains. To do this, we first calculate the *average known velocity* $v_{k}$ at each time k. This is done by defining $S_{k}$ to be the set of all outfield players (on both teams) such that their trajectories contain points at times k+1 and k, and then setting $v_{k}$ to be the average difference between their recorded points at these times. Note that if no trajectories have positions at both times k+1 and k, we set $v_{k}=0$.

Using a linear interpolation of these velocities, u(t), we can then define a weighted average velocity


(2.6) $$\begin{array}{lcr} & w(s,t)=\alpha \int_{s}^{t}u(r)dr & \end{array}$$

for some constant α. We will show later that setting α≈0.6 leads to the best results, which we believe is because players near the ball will be moving with higher velocity than players further away. Note that we use α=0.6 in our subsequent analysis.

Once the velocities have been calculated, the continuous player paths are estimated as follows. Suppose that a trajectory $T_{i}$ contains positions at times $t_{1}$ and $t_{2}$ and contains no positions in the interval $\left(t_{1},t_{2}\right)$. Then, for $t\in \left(t_{1},t_{2}\right)$*,* we set the path


(2.7) $$\begin{array}{lcr} & x_{i}(t)=x_{i}(t_{1})+w(t_{1},t)+(\frac{t-t_{1}}{t_{2}-t_{1}})(x_{i}(t_{2})-x_{i}(t_{1})-w(t_{1},t_{2})). & \end{array}$$

There are two components to this formula. Firstly, there is the simple linear interpolation between the positions, which would be the estimate if the velocity were ignored.


(2.8) $$x_{i}(t_{1})+(\frac{t-t_{1}}{t_{2}-t_{1}})(x_{i}(t_{2})-x_{i}(t_{1})).$$

Secondly, there is the velocity correction


(2.9) $$\begin{array}{lcr} & w(t_{1},t)-(\frac{t-t_{1}}{t_{2}-t_{1}})w(t_{1},t_{2}). & \end{array}$$

This is the difference between the true velocity integral up to time t and the linear approximation to it based only on the total velocity integral in the interval $\left(t_{1},t_{2}\right)$. This ensures that players follow more realistic paths as their velocities are related to those of other players. Indeed, the overall formula could be derived by considering a linear interpolation to the difference between $x_{i}$ and the ‘visible centre of mass’ (an object moving according to the visible velocity u).

### 2.7. Runtime

Using the pre-trained ARMAX model, the vast majority of the runtime for the code is performing the trajectory assignments, which runs at approximately 0.23 s/frame on a standard laptop through Jupyter Lab (Python) on a single core with a clock speed of 2.6 GHz. This means that a match with one frame sampled per second can be processed in 20−25 min, depending on the amount of stoppage time. Matches with an extra time period, of course, take proportionally longer.

## 3. Results

### 3.1. Data

We test our model using Match 1 of the Metric Sports open dataset [15]. The first half and second half are tested separately (as the players swapping sides during the interval would lead to substantial errors). Moreover, the first and last 30 frames of each half are ignored (as this contains the players entering and leaving the pitch, where they move in an unusual way and are not near the ball—again, including these frames would simply introduce an unrealistic level of errors).

To simulate the effect of broadcast data, we sample frames every second and record only players who are within 30 m of the ball. This leads to an average of 10.60 out of the 20 outfield players being visible, which is substantially less than the Statsbomb data (where on average 14.35 of the 20 outfield players were visible, though this ranged substantially from 10.93 to 18.58 depending on the match). Thus, we expect the results of this test will be robust to the change of the dataset.

There is, on average, a smaller number of available frames in the Statsbomb data—there are frames approximately every 1.9 s (rather than a frame every second in our test). However, rather than being uniformly spread, these frames are concentrated on action points in the match (where the players are moving quickly), rather than, for example, in the periods of time waiting for set pieces to be taken. With the ball in play for around 60% of football matches [16], this means that the frame rates when the ball is in play will be comparable.

Ignoring, as explained previously, the last 30 frames from each half, the total number of frames sampled is 6390, and the total number of position predictions is 59 821.

### 3.2. Error analysis

Recalling that we sample frames at a constant rate of one per second, we consider the errors in our estimation both ‘in phase’ (i.e. after an integer number of seconds) and ‘out of phase’ (i.e. after a half-integer number of seconds). We also consider the overall error (including the players for whom we know the position exactly) and for players that are ‘off-camera’—that is, the errors in estimating the positions of players with positions that were not recorded.

To calculate the error at a given time, we first map the set of estimated player positions on each team to the set of true positions using the Hungarian algorithm, where the cost is the distance between the estimated and true positions. Our error for a given player is then the distance between its estimated position and the true position to which it has been assigned.

**Figure 1.**
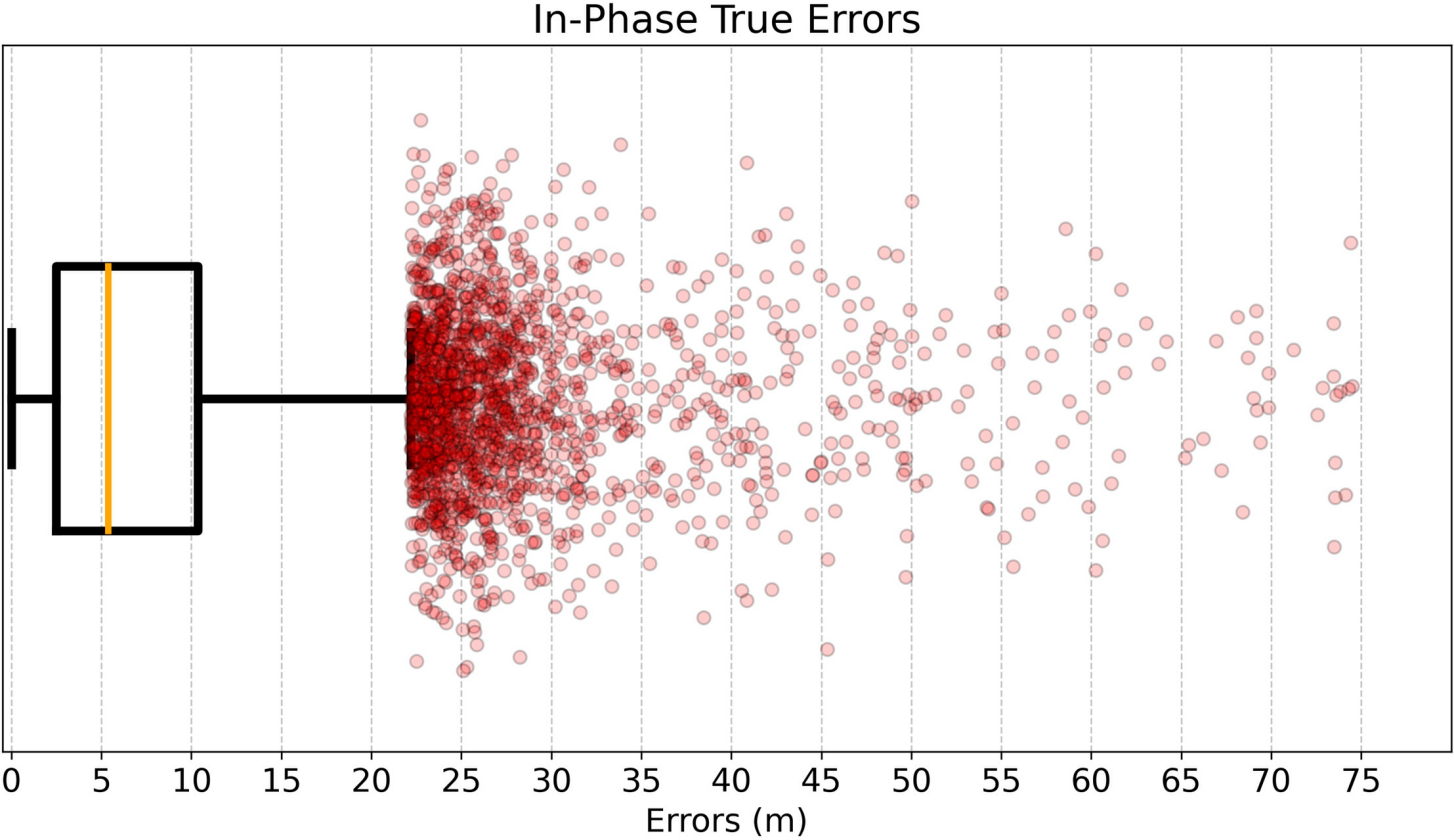
A boxplot showing the distribution of the in-phase true errors (that is, the errors for players not visible in the frame). The red dots show the distribution of errors above the 99th percentile.

When we consider the times that are in phase, many of the ‘estimates’ are exact, as, from the interpolation (2.7), the continuous player path $x_{i}(t)$ *interpolates* the player trajectory $T_{i}$. Thus, while the mean error for all players is 3.45 m, a more realistic metric is the mean error for off-camera players, which is 7.33 m. This mean is dominated by some very high errors, which can be caused, as evidenced by the fact that the median off-camera error is substantially lower at 5.37 m. The distribution of these errors is shown in figure 1.

The out-of-phase results are similar, with an overall mean error of 3.55 m. Again, this is substantially reduced by players whose positions were recently observed, highlighted by the fact that the error for players whose position was observed in the previous frame is 0.22 m.

One of the major sources of error is the frames where the ball is ‘dead’ (for example, if play has been paused because of a free kick or throw-in). This is highlighted by the fact that if only the in-phase frames closest to those in which an event has been recorded in the associated event data provided by Metrica Sports are used, the mean off-camera error drops dramatically to 6.62 m.

**Figure 2.**
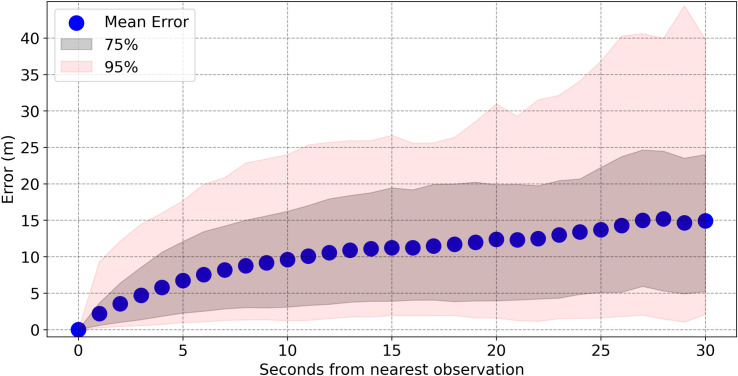
A plot showing the mean error and symmetric 75 and 95% intervals (shaded black and red regions) of the prediction errors depending on the amount of time between the prediction and the nearest observation (either forwards or backwards in time).

Unsurprisingly, the error is highly correlated with the amount of time between the prediction and the nearest observation (e.g. if a player’s trajectory contained points at times 5 and 10 s and the prediction was made at time 8 s, this time to the nearest observation would be 2 s). Figure 2 shows that the error grows relatively slowly, with players off-camera for 20 s, and therefore with a maximum of 10 s to the nearest observation still having a mean error of under 10 m and a $97.5^{\text{th}}$ percentile of less than 25 m. Given that a professional athlete could easily run between any two points on the pitch in 20 s (a maximum distance of 145 m), this shows the ability of the model to provide sensible predictions even when there are very little data.

### 3.3. Benchmarking

To put these results in context, we test our model against simple benchmarks to highlight its efficacy. Firstly, we consider benchmarks derived from our model with less effective smoothing—either where the velocity adjustment is ignored (i.e. α=0) or where we suppose that players are static between each observation. Secondly, we consider constant benchmarks—firstly using one where each player has the same position, and secondly, one where we use 10 separate positions for the outfield players of the home team (note that in the second case, to reduce the computational complexity of the calculations, we apply the optimization to a subset of 5000 frames and look at the home team only).

Table 1 shows that our model offers substantial improvement of over 70% compared to the external benchmarks, highlighting its high level of efficacy. We see that the trajectory smoothing is a crucial aspect of our model, enabling it to perform far better than both the static version and the version without velocity adjustment. This highlights that the innovations included in the model are effective at reducing the error in our predictions.

**Table 1.** A comparison of the model RMSE with other benchmarks. Note that we take the RMSE of only players that are not visible on-camera at in-phase frames, as elsewhere in this section.

benchmark	RMSE
model	7.33
model (without velocity adjustment)	9.17
model (static positions; in phase)	17.6
constant (10 positions)	25.6
constant (1 position)	43.8

### 3.4. Error variation with event type

The type of event can have a substantial effect on our model’s performance at predicting off-camera players, as summarized in table 2. To create this table, we calculated the error at the in-phase frame that was immediately before the event time, meaning that there was a time difference of at most 1 s. Note that the same frame could therefore be assigned to multiple events.

**Table 2.** A comparison of the in-phase off-camera RMSE in the frames closest to different event types, taken from the associated event data. We also display the count of these frames.

event type	count	RMSE
kick off	6	11.62
**model average (no event**)	**5157**	**7.41**
challenge	158	7.41
ball out	50	7.39
goal kick	16	7.38
**model average (all frames**)	**6390**	**7.33**
recovery	271	7.33
ball lost	246	7.25
throw in	28	7.19
card	4	7.11
**model average (any event**)	**1233**	**6.97**
pass	774	6.89
shot	24	6.84
corner kick	11	6.19
fault received	22	6.02
free kick	32	6.02

Table 2 shows that our model performs best at frames that are close to a recorded event. This is to be expected, as player behaviour becomes less predictable when there is a break in play, and therefore it is harder to forecast the off-camera movements. This improved performance close to events is encouraging for our model, as these are the parts of the match that are most likely to be of interest to analysts.

### 3.5. Error variation with α

The primary tunable parameter in our model is α, which controls the weight given to the velocity adjustment when creating the smoothed trajectories.

**Figure 3.**
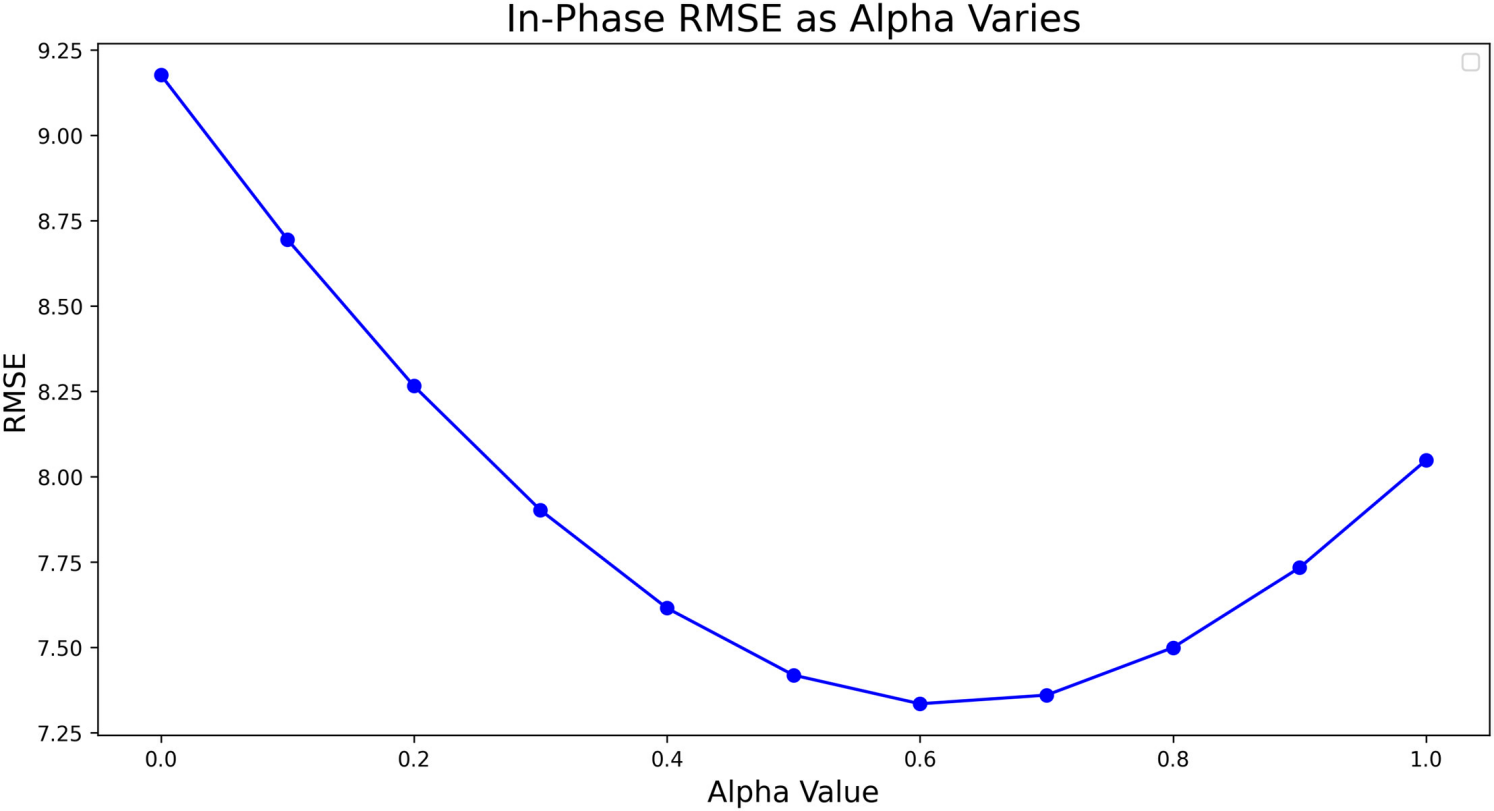
The variation of the in-phase RMSE on the test dataset for different values of α.

We see from figure 3 that the optimal value of α is approximately 0.6 (which was used in the rest of the analysis). However, there is relatively insensitive dependence around this point, with the RMSE remaining within 1% of the optimum for α=0.5 and α=0.7. When α is taken far from this range, we see that the error does begin to increase appreciably, with the extreme α=0 point being equivalent to the ‘no velocity adjustment’ benchmark discussed previously.

### 3.6. Examples

To illustrate the performance of the model, after discarding the first and last 30 frames, we choose the first frame in the first half of Match 1 where the total squared error (that is, the total sum of the squared prediction errors at that frame) is closest to the $25^{\text{th}}$, $50^{\text{th}}$, $75^{\text{th}}$ and $95^{\text{th}}$ percentile of all frame errors. This provides a fair overview of the model performance and puts these errors into the context of the match.

**Figure 4.**
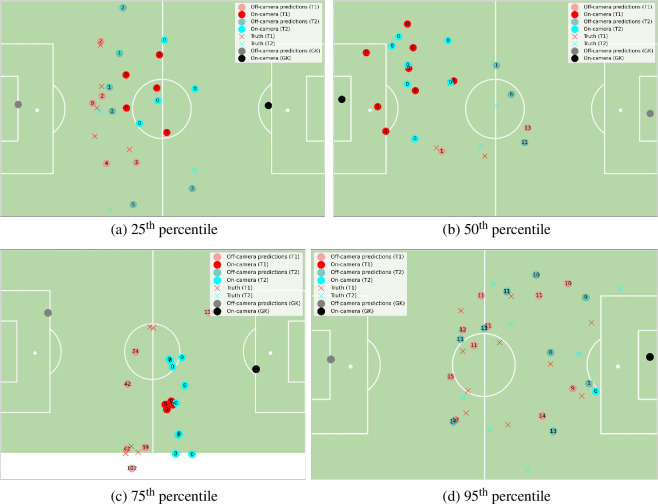
An illustration of model performance based on frames where the total squared prediction error is at different percentiles. The numbers inside each of the dots are the number of frames to the closest frame in which that player was observed (according to the trajectories created by the model). Note that goalkeepers’ predicted positions are included, but were not part of the error analysis. In all examples, the red team is attacking from left to right.

These examples are shown in figure 4. At the $25^{\text{th}}$ percentile, figure 4a provides an example where, despite the majority of the players being off-camera, the errors are small and, importantly, the overall structure of both teams remains the same. This includes one of the red central defenders (with a ‘9’ in the middle of their circle), even though the closest frame in which they are visible is 9 frames (or, equivalently, seconds) away. Being able to maintain an accurate prediction of their position despite being off-camera for a total of at least 18 s highlights the ability of our algorithm to produce useful predictions.

Figure 4b is at the $50^{\text{th}}$ percentile of total error. In this frame, the majority of players are visible, with two players (the furthest right blue and red circles) having been off-camera for a long time—at least 22 s. Their predictions are reasonably inaccurate, but importantly, the structure of both teams is still roughly correct—the red player is playing as a single striker towards the right-hand side of the pitch, while the blue defender is marking him. Thus, using these positions to aid prediction of the goalscoring potential of each team is likely to have small errors (much less so than, for example, if the predictions for these players were very far apart from each other).

Figure 4c illustrates the source of a substantial proportion of the high-error frames. Using the associated event data provided by Metrica Sports, we can see that this frame is in the middle of a 3 min long stoppage following a foul. During this time, the players move in unusual patterns (with, for example, many players grouped closely together), while players on the other side of the pitch are off-camera for an extended period of time (the rightmost red player was off-camera for around 4 min). Knowing the positions of the players during this time is much less important, as the ball is ‘dead’ and players are slowly moving towards their positions for the free kick. However, again, the overall structure is mostly maintained, illustrating the robustness of our model.

Finally, figure 4d, at the $95^{\text{th}}$ percentile of total error, is an example of the model performance when most players are off-camera for a large period of time. This occurs particularly regularly (as in this frame) at goal kicks. This leaves the model to be susceptible to error when the off-camera players move substantially while they are hidden. This was the case while the teams waited for this goal kick to be taken, as one of the pair of close-together blue players on the edge of their D (the curved region on the edge of the penalty box) moved to their right-hand side (that is, the top of the pitch as presented in the figure). Thus, the errors in team structure that are visible in this figure are much reduced by the time the goal kick is taken—indeed, the total squared error reduces from 2950 to 900 ${\text{m}}^{2}$ in the frame before the goal kick is taken (despite the fact that none of the outfield players are visible in this frame). Thus, the impact of these errors is minimal as they would have little impact on the evaluation of the game, and they do not cause any mis-assignment of player trajectories, as none of the players are visible during this time.

### 3.7. Statsbomb data

We have also produced a version of the code that can process Statsbomb’s discrete broadcast data (their ‘360 data’). This code is available at [6]. The processed data are given in the same JSON format as Statsbomb’s data, with an additional field to show whether the player was visible in the frame or whether the position has been estimated by our algorithm. A very small percentage of the frames will return an error due to large disagreements between the position of the ball in the 360 data and the associated event data, also found at [5] (these event data were used as the Statsbomb data measure its *x* coordinates from the defending team’s goal line, meaning that the axes are not fixed). These errors will be recorded in our outputted files.

## 4. Discussion

The position prediction model introduced in this paper has the potential to enrich discrete, incomplete datasets of player positions created from broadcast data. It performed well on our test dataset, achieving a mean error of 7.33 m for non-visible players, which reduced further to 6.97 m in frames that were close to a recorded event. We believe this is sufficiently accurate to be useful for a wide range of modelling tasks, as in the context of a football pitch, it is a relatively small distance (for example, a circle of radius 7.33 m would fill only 1.75% of a football pitch).

Our model compares well to the results presented in the event data model [14]. Rescaling their pitch to be the same length as ours (their pitch measured 105 m by 68 m, so all distances are multiplied by 120/105), their mean error was 7.86 m. This is over double our mean error of 3.45 m (of course, this is largely due to the fact that our model has more data) and higher than our off-camera means at events (6.97m) and at all frames (7.33m). While the difference between our off-camera prediction error and the overall error in [14] is not as large as perhaps would be expected, players closer to the ball are much easier to predict (as a triviality, the player carrying out the event can be predicted with zero error in [14] but would be excluded from our off-camera dataset), and so it seems likely that the mean error in the [14] would increase substantially if only off-camera players were included. However, including some of the features of the model in [14] could improve our prediction of player positions when they have been off-camera for a substantial amount of time.

Despite its strong performance, there remain limitations to the model. In particular, it is not designed to track individual player trajectories, with events such as corners, where many players move to a small area of the pitch, mean that our trajectory assignment algorithm often ‘swaps’ players. This could be ameliorated by, for example, adding a prior based on a player’s position within their team’s formation, which could more reliably estimate long-term player trajectories.

Moreover, our method can struggle in the period after a long stoppage in play. This may be particularly relevant with the increase in drinks breaks during matches, where play is stopped for a substantial period of time, and there is no predictable structure at the restart (so that, for example, the method used to estimate player positions at kickoff cannot be used). This could be remedied by adding a method that looked at the match in reverse, with an ensemble prediction from these two models used to increase accuracy.

Finally, while our level of error would allow, for example, teams’ attacking and defensive structures to be investigated, it would be difficult to use the estimated positions to examine attributes such as the positioning ability of off-camera players, where the impact of moving a couple of metres can be substantial. Moreover, one could not use the data to examine off-camera runs from players, as our interpolation algorithm would not be able to identify a sudden movement followed by a period of low activity. Thus, use of these data requires a careful consideration of the intrinsic error, and any analysis that requires a high level of accuracy in the player positions has the potential to produce misleading results.

Despite these limitations, we believe that our algorithm provides analysts with a substantially enriched data source that could assist in a number of tasks, both in football and, potentially, other team sports such as basketball and hockey. We believe our level of error is low enough to provide useful information about team formations and shape in different phases of the game; to train advanced expected threat models (aiming to predict the probability of the current possession ending in a goal); and to identify frequent patterns of play, such as pressing structures or common attacking movements. These advanced insights are available through data that can be manually collected, with the potential to widen the scope of advanced football analytics.

## Data Availability

The underlying data can be found at https://github.com/metrica-sports/sample-data and https://github.com/statsbomb/open-data/tree/master/data. The software developed in this paper can be found in the repository https://github.com/mpenn114/Football-Tracking-Interpolation and is archived in Zenodo here: [17].

## Appendix A. Data requirements

### Appendix A.1. Inputs

The primary processing function within the code is designed to work with Statsbomb 360 data. However, it does not require the full complexity of this data, and we present a minimal schema below.

In the Statsbomb processing pipeline, data are expected to come in two separate files—one containing the 360-style data, with the player locations at each event, and the other containing the associated event data.

Every record in the 360-style data should contain a freeze_frame, which contains the following information for each visible player:

**Table:**

field	type	description
location	[x,y] coordinates	player’s position on the pitch (scaled to 120 × 80 units). Currently, the pipeline expects these coordinates to be chosen such that (0,40) refers to the goal that the team in possession is defending
teammate	Boolean	True if the player is on the team currently performing the event
keeper	Boolean	True if the player is a goalkeeper
actor	Boolean	True if the player is the one performing the event (e.g. passing or shooting)

The associated event data should contain:

**Table:**

field	type	description
team	dictionary {name: string}	name of the team performing the event. Note that this is currently expected to follow the Statsbomb convention of being a dictionary with a field of name which gives the team’s name
period	integer	match period (1 = first half, 2 = second half, etc.)
minute	integer	game clock minute at which the event occurs
second	float	seconds within the current minute
location	[x,y] coordinates	event location on the 120 × 80 pitch

Note that there must be a method of linking the event data to the 360 data (in the Statsbomb data, this is done through the keys linked with each frame or event).

### Appendix B. Outputs

The outputs of the data are almost identical to the inputted 360-style data, with the addition of the following field:

**Table:**

field	type	description
visible	Boolean	True if this player’s position was recorded in the original data

Note that the model may return errors for individual frames where it was not able to determine the team in possession.
